# Microbial Primer: Artificial intelligence for microbiologists

**DOI:** 10.1099/mic.0.001629

**Published:** 2025-11-13

**Authors:** Hanqun Cao, Cesar de la Fuente-Nunez

**Affiliations:** 1 Machine Biology Group, Departments of Psychiatry and Microbiology, Institute for Biomedical Informatics, Institute for Translational Medicine and Therapeutics, Perelman School of Medicine, University of Pennsylvania, Philadelphia, Pennsylvania, USA; 2 Departments of Bioengineering and Chemical and Biomolecular Engineering, School of Engineering and Applied Science, University of Pennsylvania, Philadelphia, Pennsylvania, USA; 3 Department of Chemistry, School of Arts and Sciences, University of Pennsylvania, Philadelphia, Pennsylvania, USA; 4 Penn Institute for Computational Science, University of Pennsylvania, Philadelphia, Pennsylvania, USA

**Keywords:** artificial intelligence, machine learning, microbiology

## Abstract

Artificial intelligence (AI) and machine learning (ML) are reshaping microbiology, enabling rapid antibiotic discovery, resistance prediction and clinical diagnostics. For microbiologists, the goal is not to build new algorithms but to recognize when ML is appropriate, how to prepare data and how to interpret outputs responsibly. This primer takes that practical stance – driving the ML car rather than rebuilding the engine. At a high level, ML learns from complex patterns, often noisy data. In antibiotic discovery, ML models help identify compounds in biological data and design new ones from scratch using generative AI. In microbiome studies, where measurements are compositional, sparse and often confounded, ML helps uncover community structure and link taxa or functions to phenotypes. In pathogen genomics, supervised models map sequence-derived features (e.g. k‑mers, SNPs and gene presence/absence) to outcomes such as species identity, antimicrobial susceptibility or MIC. Unsupervised learning supports exploration, including clustering, latent gradients and dimensionality reduction for visualization. Across these settings, success hinges less on exotic architectures than on sound problem framing, careful preprocessing and experimental validation.

## Introduction

Artificial intelligence (AI) and machine learning (ML) are reshaping microbiology, enabling rapid antibiotic discovery, resistance prediction and clinical diagnostics. For microbiologists, the goal is not to build new algorithms but to recognize when ML is appropriate, how to prepare data and how to interpret outputs responsibly [1]. This primer takes that practical stance *– driving* the ML car rather than rebuilding the engine. At a high level, ML learns from complex patterns, often noisy data [2]. In antibiotic discovery, ML models help identify compounds in biological data and design new ones from scratch using generative AI. In microbiome studies, where measurements are compositional, sparse and often confounded, ML helps uncover community structure and link taxa or functions to phenotypes. In pathogen genomics, supervised models map sequence-derived features (e.g. kmers, SNPs and gene presence/absence) to outcomes such as species identity, antimicrobial susceptibility or MIC. Unsupervised learning supports exploration, including clustering, latent gradients and dimensionality reduction for visualization. Across these settings, success hinges less on exotic architectures than on sound problem framing, careful preprocessing and experimental validation.

**Scope of this primer.** We (i) outline core ML task families (i.e. classification, regression, clustering and dimensionality reduction) and map them to common laboratory questions; (ii) summarize data representations and preprocessing that respect measurement constraints (e.g. centred log- ratio transforms for compositional data; phylogeny-aware encodings for genomes; signal handling for spectra, images and time series); (iii) provide model selection heuristics that start with interpretable baselines and scale to ensembles or deep learning when justified; and (iv) detail validation designs that mirror deployment (stratified, temporal/geographic and subject-level splits) and metrics robust to class imbalance. We also introduce model interpretation for biologically testable hypotheses and illustrate principles with use cases in antibiotic discovery, resistance prediction (sequence- and MALDI-based), rapid diagnostics and immunology. Throughout, we flag common pitfalls (e.g. data leakage, batch effects and overfitting) and include concise, practical checklists.

## ML primer for microbiologists

### Basic definitions

**Fig. 1.**
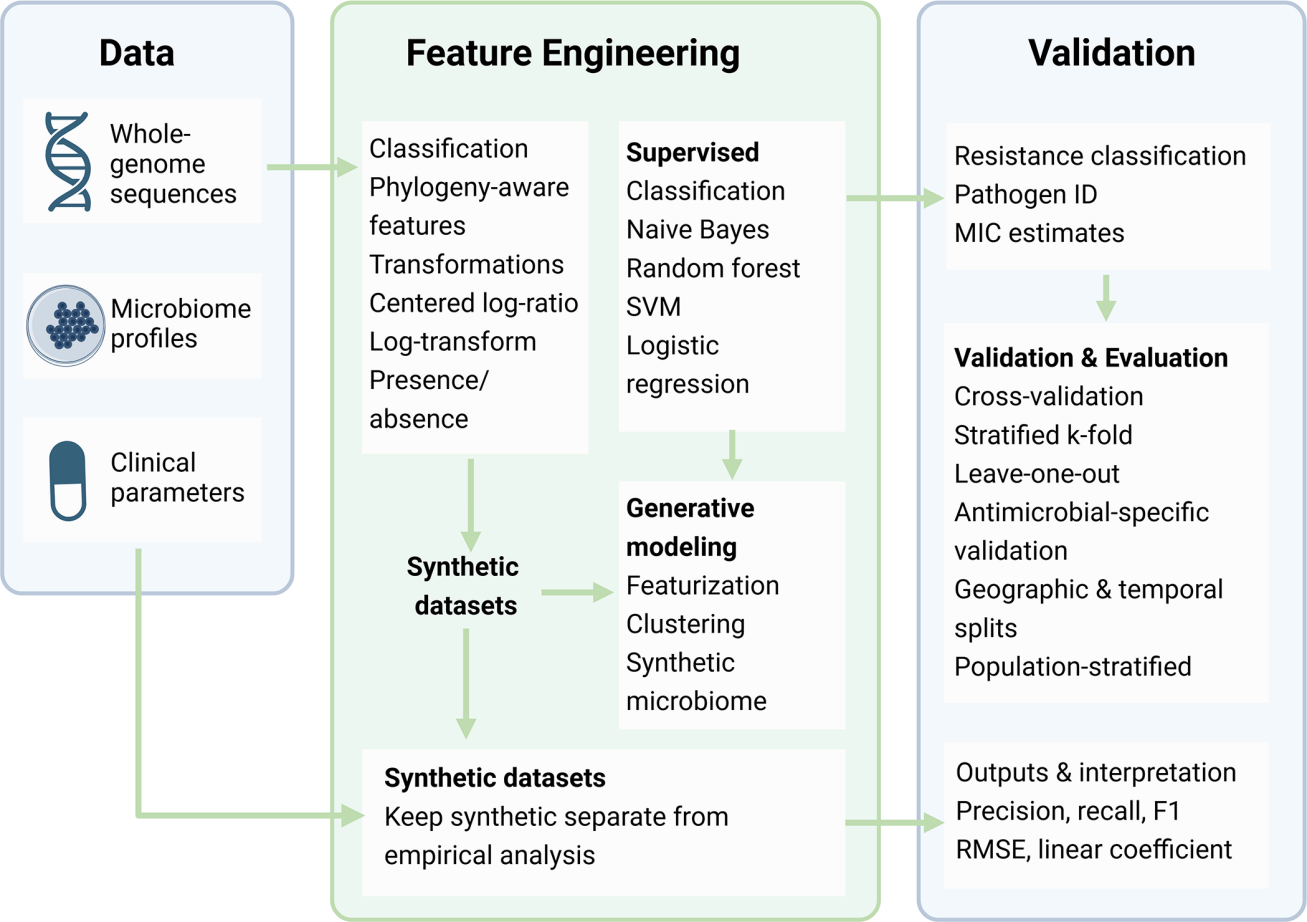
This AI/ML workflow transforms diverse microbiology inputs – whole-genome sequences, microbiome profiles and clinical parameters – into model-ready features through phylogeny-aware encodings, centred log-ratio transforms and presence/absence matrices. The framework employs supervised learners including naive Bayes for 16S data, random forest, SVM and logistic regression, alongside generative models like MiSDEED and MB-GAN. Synthetic data remain isolated from empirical analyses. Validation strategies mirror real-world deployment through stratified cross-validation, leave-one-out approaches and geographic, temporal and population-based splits. The system delivers resistance classifications, pathogen identification, MIC estimates and performance metrics. (Created with BioRender.com.)

**ML systems** are computational frameworks that learn predictive relationships from data without explicitly programming those relationships (Fig. 1). Unlike conventional statistical approaches that require predefined distributional assumptions, these algorithms derive predictive models directly from empirical observations. In antimicrobial research, ML systems can be used to analyse complex datasets including whole-genome sequences, microbiome profiles and chemical structures to predict antibiotic susceptibility, identify resistance mechanisms and guide therapeutic decisions without requiring prior knowledge of biological pathways.

**Models** represent mathematical functions that map input features to predictive outcomes through parameter optimization during training phases. In antibiotic applications, for instance, models can predict resistance phenotypes from genomic markers, classify pathogens from sequence data or estimate MICs from molecular descriptors. These models learn by minimizing prediction errors on validated datasets before deployment on novel cases.

**Data types** in antimicrobial ML encompass compositional microbiome data (normalized abundances), sequence information (genomic, transcriptomic and proteomic) and high-dimensional feature vectors (gene presence/absence, chemical properties and spectral signatures). Compositional data present unique analytical challenges due to mathematical constraints in proportional measurements, necessitating specialized preprocessing approaches that traditional statistical methods cannot adequately address.

#### Supervised learning

Supervised learning algorithms establish mappings between input features and validated outcomes, forming the foundation of predictive antimicrobial applications. These algorithms utilize training datasets containing input variables (e.g. genomic sequences, chemical descriptors and clinical parameters) paired with target outcomes, such as resistance classifications, MICs and pathogen identities, to generate predictive models for novel samples.

**Classification** predicts discrete labels (e.g. species ID; resistant vs. susceptible) from input features. It constitutes the primary supervised approach in antimicrobial research. Taxonomic identification employs naive Bayes algorithms on 16S rRNA sequences for bacterial classification, while resistance prediction utilizes random forest algorithms achieving >90% accuracy across multiple species when applied to genomic features. Support vector machines excel in binary resistance/susceptibility determinations, whereas logistic regression provides interpretable biomarker identification through coefficient analysis.

**Regression** predicts continuous values (e.g. MIC, growth parameters and dose–response). Temporal regression can model microbiome dynamics after antibiotic exposure.

#### Unsupervised learning

Unsupervised methods find structure in unlabeled data.

**Clustering** groups unlabeled samples into similarity-based clusters without using outcome labels. It is used in the microbial sciences, for example, to stratify patients by microbiome composition for personalized antimicrobial therapy and identify co-occurring bacterial communities suggesting synergistic resistance mechanisms. Network analysis reveals microbial interactions relevant to antibiotic efficacy and resistance development.

**Dimensionality reduction** [e.g. Principal Component Analysis (PCA), t-distributed Stochastic Neighbor Embedding (t-SNE) and Uniform Manifold Approximation and Projection (UMAP)] projects high-dimensional data into low-dimensional spaces for visualization and pattern discovery (e.g. resistance gradients and lineage structure). While mapping high-dimensional data to a lower-dimensional space, this method preserves salient structure (variance, distances or neighbourhoods). It enables the visualization of high-dimensional datasets through PCA applied to phylogenetic distance matrices (UniFrac) or compositional dissimilarity measures (Bray–Curtis). Advanced nonlinear methods, including t-SNE and UMAP, reveal complex resistance patterns invisible to linear approaches.

#### Feature engineering

**Feature engineering** translates biological data into algorithm-compatible formats and often determines performance more than algorithm choice. Effective implementation requires integration of microbiological domain knowledge with computational constraints.

**Taxonomic integration** exploits hierarchical classification systems by creating multi-scale features spanning species-to-phylum levels, enabling pattern detection across biological scales. Phylogeny-aware feature design encodes evolutionary relatedness (e.g. phylogenetic eigenvectors or PCA coordinates, lineage one-hot/embeddings or kernels built from patristic distances) to respect shared ancestry.

**Data transformations** address compositional constraints through centred log-ratio transformation and manage abundance distributions via log-transformation. Presence-absence encoding occasionally matches abundance-based performance, suggesting community composition primacy over precise quantification in resistance prediction.

**Feature selection** employs differential abundance analysis, multivariate signature identification and embedded regularization methods (LASSO) to maintain biological relevance while reducing dimensionality for robust antimicrobial prediction models.

### Generative modelling

Generative models learn data distributions to synthesize realistic examples, addressing data scarcity and enabling *in silico* experimentation.

**Synthetic microbiomes.** Tools such as MB-GAN generate plausible abundance profiles; ecological simulators (e.g. Lotka–Volterra-based) produce longitudinal trajectories (e.g. MiSDEED). These aid power analysis, algorithm validation with ground truth and robustness checks.

**Peptide/drug design.** Variational autoencoders, diffusion models and protein language-model-based generators propose novel antimicrobial peptides or small molecules for experimental testing.

**Good practice.** Keep synthetic and empirical datasets separate in analyses to avoid biased conclusions.

#### Model selection and evaluation

**Overfitting** occurs when models learn training data patterns too specifically, resulting in poor generalization to new data despite excellent training performance. In antimicrobial resistance (AMR) prediction, for example, overfit models may show excellent development performance but fail when deployed clinically, potentially causing treatment failures. Regularization techniques (L1/L2 penalties, dropout) and feature selection strategies can be used to address the curse of dimensionality inherent in whole-genome sequencing (WGS) data.

**Cross-validation** represents a resampling procedure that estimates generalization by repeatedly splitting data into training and validation folds, ultimately preventing overfitting in high-dimensional datasets. In k-fold cross-validation, data are partitioned into k subsets, and each serves once as the validation set, which ensures balanced resistance phenotype representation, while leave-one-out approaches accommodate small clinical cohorts. Microbiome studies require careful consideration of temporal and familial dependencies that violate standard independence assumptions.

#### Deployment-mirrored validation

- **Geographic splits:** train on some hospitals, test on others.
- **Temporal splits:** train on earlier years, test on later cohorts.
- **Population-stratified splits:** ensure performance across demographics.

#### Evaluation metrics

- **Classification:** precision, recall, F1, AUROC and AUPRC (often more informative than accuracy under class imbalance).
- **Regression:** RMSE and MAE.
- **Calibration and uncertainty:** quantify reliability of predicted probabilities for clinical use.

#### Model interpretation

**Feature importance analysis** identifies microbial taxa and genomic elements driving antimicrobial predictions, generating hypotheses about resistance mechanisms. SHAP (SHapley Additive exPlanations, a game-theoretic feature attribution method) values provide individual prediction explanations, elucidating how specific features contribute to resistance classifications or MIC estimates. Linear models offer direct coefficient interpretation for biomarker discovery, facilitating identification of clinically actionable antimicrobial indicators despite potentially reduced predictive performance compared to ensemble methods.

## Applications of ML in microbiology

**Antibiotic discovery:** Computational and AI approaches have revolutionized antibiotic discovery. For example, Porto *et al*. [3] applied evolutionary programming to design guavanin-2, a synthetic peptide capable of reducing infections in mouse models, which targeted bacteria through an unusual hyperpolarization mechanism of action. In another landmark paper [4], graph neural networks identified halicin from over 100 million chemical molecules, revealing potent activity against multidrug-resistant pathogens including *Mycobacterium tuberculosis* and carbapenem-resistant *Enterobacteriaceae*. Moreover, Wong *et al*. [5] proposed an explainable graph neural network to identify novel antibiotics from over 12 million compounds, successfully discovering new structural classes.

The field has expanded into mining biology [6]. Indeed, several AI models, including ensemble neural networks combining LSTM, attention mechanisms and transformer architectures, have been leveraged to mine antimicrobial peptide candidates from metagenomes. These computational approaches identified thousands of small ORF-encoded peptides, including prevotellin-2, SCUB1-SKE25 and many other promising candidates from proteomes, such as archaeasins [7].

Advances have also been made in generative AI. For example, HydrAMP used deep generative models for novel AMP creation with 96% experimental success rates, while foundation models like deepAMP achieved over 90% success in broad-spectrum antimicrobial peptide design. Large language models further advanced the field, with AMP Designer demonstrating 94.4% success rates through prompt tuning approaches. Bayesian optimization methods are applied in ApexGo to enhance antimicrobial properties.

AI approaches have targeted specific challenges in antibiotic resistance. For instance, a recent computational exploration of global venoms (the Venomics AI project) used APEX [8] to identify novel antimicrobial scaffolds from over 40 million peptide compounds, while ApexDuo addressed intracellular infections by designing dual-function peptides with both cell-penetrating and antimicrobial properties. An advanced framework like ApexOracle extends discovery to non-canonical targets and unknown pathogens. Furthermore, advanced platforms, such as ApexAmphion, extend antimicrobial discovery from 10,000 orders of magnitude to 1,000,000 orders of magnitude with pretrained large protein language models and reinforcement learning. This progression demonstrates AI’s transformation from a screening tool to a comprehensive design platform capable of addressing complex AMR challenges.

### Resistance prediction

ML is also being employed to predict AMR directly from genomic and clinical data, bypassing traditional, time-consuming resistance testing methods. Through the analysis of WGS data, ML models can predict resistance profiles with over 90% accuracy, helping clinicians make faster, more accurate treatment decisions. For example, DeepARG, a deep learning-based tool, can predict resistance genes in bacterial genomes, making it highly effective in resistance classification across various pathogens and antibiotics. This system is particularly useful in clinical settings, where the quick identification of resistance mechanisms is vital for appropriate antimicrobial therapy.

Beyond genomic data, MSDeepAMR is [9] another groundbreaking model that uses MS data to predict resistance phenotypes. This system requires no manual preprocessing and allows for near-instantaneous identification of resistance patterns from bacterial MS data, improving the diagnostic speed and accuracy compared to traditional methods. The ability to predict AMR from mass spectra directly reduces the reliance on culture-based assays, which are typically slow and labour-intensive.

Beyond genomic and proteomic features, ML can improve the phylogenetically robust prediction of AMR by explicitly modelling ancestry. Practical strategies include lineage-aware evaluation (e.g. leave-one-lineage/clade/site-out splits to avoid ancestry leakage), hierarchical or mixed-effects models with random intercepts/slopes for lineage and collection site to absorb population structure and phylogeny-derived similarity priors (tree-based kernels/graph Laplacians) that encode shared evolutionary history. Convergence-aware bGWAS/ML frameworks (e.g. treeWAS, hogwash and pyseer) further reduce spurious associations driven by clonal background while retaining true, repeatedly gained resistance variants. Collectively, these phylogeny-aware designs curb ancestry-driven inflation and demonstrably improve external validity – yielding higher accuracy on held-out lineages, sites or time windows in clonally structured pathogens.

### Diagnostics

**Real-time detection technologies** employ rapid biological feature analysis to address time-sensitive diagnostic needs. AI-enhanced spectroscopic methods, including Raman spectroscopy and MALDI-TOF MS, utilize deep learning algorithms for pathogen identification and resistance phenotype prediction. Digital PCR with high-resolution melt curve analysis enables simultaneous pathogen identification and antimicrobial susceptibility testing. Additionally, microscopy-based approaches incorporating ML analyse bacterial growth patterns and movement for rapid susceptibility determination, while microfluidics platforms accelerate traditional testing workflows.

**Genomic prediction technologies** resolve complex mechanisms beyond known markers. Rules-based genotyping remains effective for catalogued ARGs, while pan-genome ML (unitigs/k-mers/graphs) learns genome-wide features to predict MIC/phenotype and surface novel loci/pathways shared across drugs or lineages. Multi-omic integration (WGS+transcriptome) improves phenotype mapping where expression modulates resistance. For metagenomes and culture-independent contexts, DeepARG and related deep models reduce false negatives versus best-hit heuristics and generalize to divergent ARG families. Together, these approaches expand from ‘is the gene present?’ to ‘which genome-wide patterns explain resistance and are portable across cohorts?’

**Clinical decision technologies** use EHR-scale variables (demographics, comorbidities, prior microbiology/antibiotic use and care setting) to estimate pathogen and resistance pre-test probabilities and to optimize empiric therapy when lab results are pending or unavailable. Such models consistently improve discrimination for ESBL/MRSA risk and narrow broad-spectrum use in simulations and retrospective validations, offering practical guidance in resource-limited settings while formal prospective impact evaluations continue to grow.

### Immunology

ML is making a significant impact in immunology, particularly in vaccine design and microbiome-immune system interaction research. Tools like DeepVacPred are helping accelerate vaccine development by predicting T-cell epitopes, which are key to developing vaccines that stimulate the immune system effectively. For example, DeepVacPred was instrumental in designing multi-epitope vaccines for SARS-CoV-2, enabling rapid and effective responses to emerging infectious diseases. These models are also being used to design vaccines for other challenging pathogens like HIV and influenza, reducing the time required for candidate identification from months to minutes.

Another significant advancement comes from AlphaFold2, which predicts the 3D structures of proteins with unprecedented accuracy. This has been particularly useful for structure-based vaccine design, such as stabilizing antigens used in mRNA vaccines. AlphaFold 2 has played a pivotal role in improving the design of spike proteins for COVID-19 vaccines, making them more effective by stabilizing their pre-fusion conformation.

In microbiome research, ML models are being used to understand how microbial communities influence immune responses, with a focus on how the gut microbiome impacts inflammation, immune modulation and disease progression. For example, AI models have linked specific gut microbiome compositions with immune responses, helping identify new therapeutic targets for autoimmune diseases and cancers. These models can analyse multi-omic data, including microbiome composition, gene function and metabolite profiles, to predict immune outcomes and improve the efficacy of immunotherapy.

## Practical considerations for microbiologists

For microbiologists adopting ML tools, it is crucial to understand which algorithms and approaches work best for different types of data [10]. Random forests and k-mer-based methods are often effective for microbial classification and resistance prediction, especially when the dataset is relatively small or sparse. However, deep learning approaches such as convolutional neural networks (CNNs) and recurrent neural networks excel with large, complex datasets. Understanding how to preprocess and transform data is key to the success of ML models. For instance, log-ratio transformations are required for compositional microbial data to account for the constant-sum nature of such datasets, which traditional statistical methods cannot adequately handle.

In addition to selecting the right model, handling class imbalance is a common issue, particularly in clinical datasets where resistant strains are often underrepresented. Techniques like SMOTE (Synthetic Minority Over-sampling Technique, which interpolates new minority-class samples to mitigate class imbalance) or stratified sampling can help balance the training data, ensuring that the model is trained to recognize both resistant and susceptible strains. Furthermore, ML tools are becoming increasingly accessible, with platforms like HydrAMP and ADMET-AI offering user-friendly interfaces that allow microbiologists with minimal computational experience to leverage powerful ML tools for peptide design and pharmacokinetic property prediction.

Below, we delineate guidelines any microbiologist can follow when thinking about applying ML in their specific project:

### Choosing methods

- Small/sparse datasets → start with interpretable models (logistic regression, random forests and gradient boosting).
- Large/complex data (images, long sequences) → consider deep learning (CNNs, transformers) with careful regularization and interpretation.

### Preprocessing essentials

- Use CLR for compositional microbiome data;
- Normalize and batch-correct spectra/images;
- Encode sequences via k-mers or learnt embeddings.

### Class imbalance

- Use stratified sampling, cost-sensitive training or oversampling (e.g. SMOTE).
- Report precision/recall/F1 and confusion matrices, not accuracy alone.

### Tooling

- User-friendly platforms for peptide design and ADMET prediction lower barriers.
- General ML stacks (e.g. scikit-learn) and microbiome pipelines (e.g. QIIME 2) integrate well with bench workflows.

### Quick-start checklist

1. Define the biological question and outcome.
2. Map inputs to features; document preprocessing.
3. Split data to mirror deployment; prevent leakage.
4. Start simple; record baselines.
5. Tune fairly with cross-validation.
6. Report appropriate metrics with confidence intervals.
7. Interpret features; sanity-check with domain knowledge.
8. Validate externally; plan for monitoring drift after deployment.

## Pitfalls

Despite the promising applications, microbiologists must be aware of potential pitfalls. Overfitting is a common challenge, especially with small datasets. Therefore, it is critical to validate models on external datasets to ensure their generalizability across different populations and settings. Data leakage can also result in overoptimistic performance estimates, so ensuring that training and test data are strictly separated is essential. Moreover, interpretability is key in clinical applications where decisions based on ML predictions could have significant implications. Researchers and clinicians must ensure that the ML models used are not only accurate but also explainable, particularly in high-stakes environments like healthcare.

## Outlook

The integration of AI into microbiology stands at an inflection point where research tools are rapidly transitioning to preclinical and clinical applications. Foundation models – large-scale AI systems trained on diverse datasets – promise to revolutionize the field further by providing general-purpose tools adaptable to specific microbiological tasks. The success in generating antimicrobial peptides and predicting protein structures suggests that similar approaches will soon enable automated hypothesis generation and experimental design in microbiology.

Regulatory frameworks are evolving to accommodate AI/ML medical devices, with several diagnostic platforms approaching FDA approval and clinical deployment. The convergence of rapid diagnostics, automated antimicrobial susceptibility testing and AI-powered clinical decision support systems will likely transform infection management from reactive treatment to predictive, personalized approaches. Multi-modal integration – combining genomic, phenotypic, clinical and environmental data – represents the next frontier, promising a more comprehensive understanding of AMR emergence and transmission patterns.

However, significant challenges remain. The need for diverse, high-quality training datasets becomes more critical as AI systems achieve broader deployment, particularly ensuring representation across global populations and healthcare settings. Explainability and interpretability of AI predictions will prove essential for clinical acceptance and regulatory approval. Perhaps most importantly, the integration of AI tools into existing laboratory workflows requires careful consideration of human factors, training requirements and quality assurance protocols to ensure that technological advances translate into accelerated scientific discovery and improved patient outcomes rather than introducing new sources of error or bias.

## Abbreviations

AI: artificial intelligence
AMR: antimicrobial resistance
CNNs: convolutional neural networks
ML: machine learning
PCA: Principal Component Analysis
SMOTE: Synthetic Minority Over-sampling Technique
t-SNE: t-distributed Stochastic Neighbor Embedding
UMAP: Uniform Manifold Approximation and Projection
WGS: whole-genome sequencing
